# From bench to bedside: murine models of inherited and sporadic brain arteriovenous malformations

**DOI:** 10.1007/s10456-024-09953-5

**Published:** 2025-02-03

**Authors:** Ashely R. Ricciardelli, Gael Genet, Nafiisha Genet, Samuel T. McClugage, Peter T. Kan, Karen K. Hirschi, Jason E. Fish, Joshua D. Wythe

**Affiliations:** 1 Department of Neurosurgery, Baylor College of Medicine, Houston, TX 77030 USA; 2 Department of Cell Biology, University of Virginia School of Medicine, Charlottesville, VA USA; 3 Robert M. Berne Cardiovascular Research Center, University of Virginia School of Medicine, Charlottesville, VA USA; 4 Division of Pediatric Neurosurgery, Texas Children’s Hospital, Houston, TX USA; 5 Department of Neurosurgery, University of Texas Medical Branch, Galveston, TX 77598 USA; 6 Developmental Genomics Center, University of Virginia School of Medicine, Charlottesville, VA USA; 7 Toronto General Hospital Research Institute, University Health Network, Toronto, ON Canada; 8 Department of Laboratory Medicine & Pathobiology, University of Toronto, Toronto, ON Canada; 9 Peter Munk Cardiac Centre, University Health Network, Toronto, ON Canada; 10 Department of Neuroscience, University of Virginia School of Medicine, Charlottesville, VA USA; 11 Brain, Immunology, and Glia Center, University of Virginia School of Medicine, Charlottesville, VA USA

**Keywords:** Cerebrovascular, Intracranial hemorrhage, AVM, RAS/MAPK, Notch, BMP, TGF-β

## Abstract

Brain arteriovenous malformations are abnormal vascular structures in which an artery shunts high pressure blood directly to a vein without an intervening capillary bed. These lesions become highly remodeled over time and are prone to rupture. Historically, brain arteriovenous malformations have been challenging to treat, using primarily surgical approaches. Over the past few decades, the genetic causes of these malformations have been uncovered. These can be divided into (1) familial forms, such as loss of function mutations in TGF-β (BMP9/10) components in hereditary hemorrhagic telangiectasia, or (2) sporadic forms, resulting from somatic gain of function mutations in genes involved in the RAS-MAPK signaling pathway. Leveraging these genetic discoveries, preclinical mouse models have been developed to uncover the mechanisms underlying abnormal vessel formation, and thus revealing potential therapeutic targets. Impressively, initial preclinical studies suggest that pharmacological treatments disrupting these aberrant pathways may ameliorate the abnormal pathologic vessel remodeling and inflammatory and hemorrhagic nature of these high-flow vascular anomalies. Intriguingly, these studies also suggest uncontrolled angiogenic signaling may be a major driver in bAVM pathogenesis. This comprehensive review describes the genetics underlying both inherited and sporadic bAVM and details the state of the field regarding murine models of bAVM, highlighting emerging therapeutic targets that may transform our approach to treating these devastating lesions.

## Introduction

**Fig. 1:**
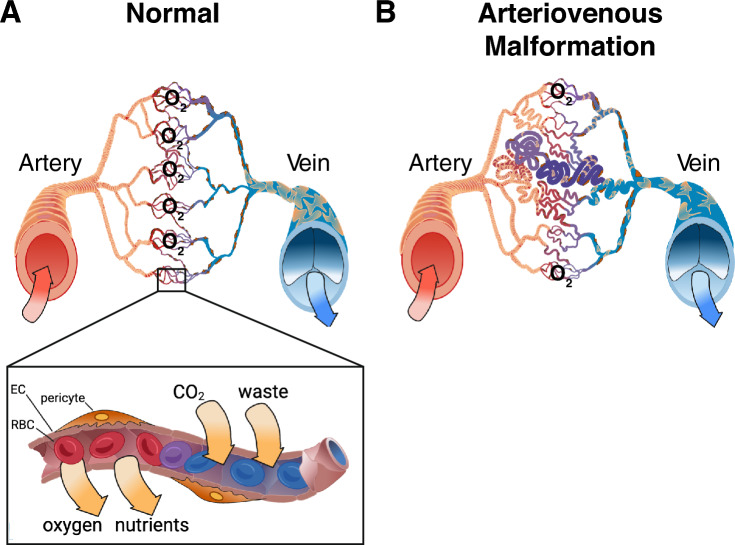
Arteriovenous Malformations. **A** Schematic of a normal arteriovenous network, where arteries and veins only communicate at the intervening capillary bed, where diffusion of oxygen, nutrients, and hormones to surrounding tissues occurs (such as the brain parenchyma). **B** An arteriovenous malformation [AVM] is the result of a direct connection between a high-flow arterial vessel and a low resistance capacitance venous vessel, with a demarcated nidus (or tangle), and extensive dilation of the draining veins, and tortuous remodeling of both the feeding arteries and draining veins. These lesions can lead to decreased flow through the surrounding capillary vessels (the “steal” phenomenon), and results in local hypoxia surrounding the AVM due to decreased capillary perfusion of adjacent regions. EC = endothelial cell; RBC = red blood cell

Arteriovenous malformation [AVM] results from an abnormal connection between an artery and a vein, creating a high flow, low resistance shunt that completely or partially bypasses the capillary network and remodels into a tangled and torturous connection between the two vessels, called a nidus [1] (Fig. 1). The resulting abnormal vascular network associated with an AVM can result in local tissue hypoxia surrounding the shunt, driving functional deficits due to decreased blood flow through the capillary vasculature, which limits delivery of oxygen and nutrients to surrounding tissues. A nidus can have a single dominant feeder artery (direct arteriovenous fistula), or multiple feeding arteries, coursing directly into the nidus or indirectly from nearby en passage vasculature. The nidus can evolve over time into a tangled mass of fragile vessels. This article will focus on brain AVMs (bAVMs), a particularly complicated and difficult to treat vascular anomaly that can lead to intraparenchymal damage due to hemorrhage, predisposing patients to significant and permanent neurological disability or death.

The current asymptomatic incidence of these cerebrovascular anomalies is estimated to be around 50 individuals per 100,000 (95% confidence interval [CI], 0.01–0.10) [2] with a detected prevalence of around 10–18 per 100,000 people, leading to an incidence of 1.3 per 100,000 person-years [3]. These lesions are responsible for up to 50% of hemorrhagic strokes in the pediatric population [4] and 6–9% in adults [5]. Clinical manifestations of bAVM include intracranial hemorrhage (around 50% of patient cases), and neurological deficits including, but not limited to headaches, focal and generalized seizures, and progressive neurological deficits [6].

Previously unruptured bAVMs present with a 1–3% hemorrhage rate per year [7–11], but this rate increases to 5% per year after a bAVM has ruptured [12]. Mortality after bAVM rupture and hemorrhage is estimated between 12 and 66.7% [13, 14] while 23–40% of survivors have significant neurological disability [15]. Managing these cerebrovascular anomalies requires intensive resources, and their treatment is technically challenging, as they often require multiple invasive treatment modalities (e.g., microsurgical resection, stereotactic radiosurgery, or endovascular embolization), further increasing the risk of stroke and hemorrhage [16]. For an expanded discussion on clinical management of these lesions, we refer readers to Ricciardelli et al. 2023 [17]. Further complications arise from the fact that even in cases of successful surgical intervention, there is a risk of recurrence, particularly in the pediatric population [18]. However, many bAVM patients are not even eligible for surgery due to the inherit risks associated with these procedures, highlighting the need for developing non-surgical interventional strategies. Accordingly, in vivo validation studies in animal models are an essential step in the identification and preclinical validation of these alternative therapeutic strategies for managing bAVM.

Most of our knowledge of the molecular mechanisms underlying bAVMs has been gleaned from the study of hereditary genetic syndromes that may feature these anomalies. However, in these inherited diseases, AVMs frequently arise in organs other than the brain (such as the spine, liver, lungs, or gastrointestinal system). Moreover, while these inherited genetic syndromes may feature multiple arteriovenous malformations, the majority of bAVMs are de novo, sporadic lesions, and only 5% are attributed to autosomal dominant disorders [19]. Thus, until relatively recently, whether there was a common etiology underlying these cerebrovascular anomalies was unclear. Below, we discuss common genetic syndromes that feature bAVMs, as well as the recent identification of somatic mutations associated with sporadic bAVM, and current murine models of both diseases. Finally, we detail current non-surgical therapeutic approaches to ameliorate these devastating vascular anomalies and highlight key remaining questions in the field.

## Congenital syndromes featuring bAVMs

### Hereditary hemorrhagic telangiectasia (HHT)

Approximately 5% of bAVM lesions are attributed to various inherited syndromes, most notably the rare autosomal dominant genetic disorder hereditary hemorrhagic telangiectasia (HHT) (Table 1) [20]. HHT, also known as Osler-Weber-Rendu disease, is characterized by recurrent epistaxis (nosebleeds), systemic telangiectasias (dilated postcapillary venules directly connected with dilated arterioles—relatively small arteriovenous malformations) on mucocutaneous surfaces, such as the skin, nasal mucosa, or lining of the GI tract, as well as larger arteriovenous malformations in internal organs such as the liver, lung, spine, and brain [21, 22]. While symptoms vary between HHT patients (even in the same family), disease penetrance is estimated at 95% by adulthood [23], with an incidence between 1:8000 to 1:5000 [24, 25].

**Table 1:** Congenital Syndromes Featuring Arteriovenous Malformations

Gene	Protein	Protein type	Normal function	Mutation	Phenotype	Clinical entity	References
*ENG*	Endoglin	Transmembrane glycoprotein TGF-β receptor III	TGF-β receptor complex member	Truncations in extracellular domain	Epistaxis, telangiectasis, high frequency of PAVM, CAVM	HHT1	McAllister et al. (1995)
*ACVRL1*	Activin receptor-like kinase 1 (ALK1)	Receptor serine threonine kinase TGF-β receptor II	TGF-β receptor complex member that phosphorylates R-SMADs	Missense variants	Epistaxis, telangiectasis, HAVM, lower frequency of PAVM and CAVM	HHT2	Johnson et al. (1996)
2 unknown genes on Chr. 5	Unknown	Unknown	Unknown	Unknown	Epistaxis, telangiectasias, PAVM, CAVM	HHT3	Cole et al. (2005)
2 unknown genes on Chr. 7	Unknown	Unkown	Unknown	Unknown	Epistaxis, telangiectasias, PAVM, CAVM	HHT4	Bayrak-Toydemir et al. (2006)
*GDF2*	BMP9	Cytokine	Ligand for the TGF-β pathway	Missense variants	Epistaxis, telangiectasias, CM	HHT5	Wooderchak-Donahue et al. (2013)
*MADH4*	SMAD4	Transcription Factor	Translocates to nucleus with R-SMADs to control transcription	Carboxy-terminal MH2 domain	Epistaxis, telangiectasias, PAVM, CAVM, HAVM, hemartomatous polyps in GI tract	JP-HHT	Gallione et al. (2004)
Unkown	Unknown	Unknown	Unknown	Unknown	Retinal AVMs, bAVMs, and vascular facial skin changes and lesions	WMS	Schmidt et al. (2008)

*HHT* hereditary hemorrhagic telangiectasia, *JP-HHT* juvenile polyposis hereditary hemorrhagic telangiectasia, *WMS* Wyburn-Mason syndrome, *PAVM* pulmonary arteriovenous malformation, *CAVM* cerebral arteriovenous malformation, *HAVM* hepatic arteriovenous malformation, *CM* capillary malformation

**Fig. 2:**
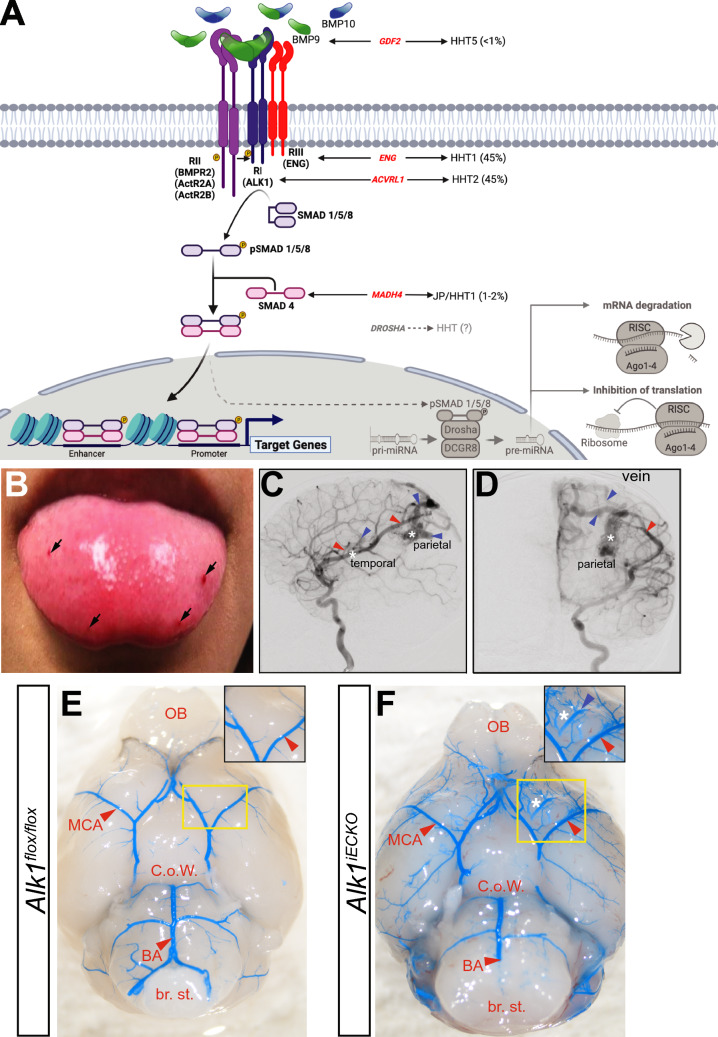
The molecular and genetic etiology of Hereditary Hemorrhagic Telangiectasia (HHT). **A** HHT is an autosomal dominant inherited syndrome that results from impaired transforming growth factor-β (TGF-β) signaling. Mutations have been observed in genes encoding multiple proteins of this pathway. Loss of function variants in *GDF2*, which encodes the ligand bone morphogenetic protein 9 (BMP9) lead to HHT type 5 (HHT5) and account for < 1% of all HHT cases, while loss of function mutations in the genes *ACVLR1* and *ENG*, which encode the TGF-β co-receptors activin receptor-like kinase 1 (ALK1) (HHT2) and endoglin (ENG) (HHT1), together account for approximately 90% of all cases of HHT. Lesions in *MADH4,* which encodes the transcription factor SMAD4, underly juvenile polyposis and HHT (JP-HHT) and are found in 1–2% of all HHT cases. A single study recently reported HHT like symptoms in patients harboring suspected pathogenic, damaging variants in the class 2 double stranded RNA-specific endoribonuclease *DROSHA*, which executes the initiation step of microRNA processing within the nucleus, and may specifically regulate a class of BMP-target microRNAs. **B** In HHT patients, small microscopic AVMs, known as telangiectasias (indicated by the black arrows), present on mucosal membranes, such as the finger nails or tongue. **C** Lateral view of a digital subtractive angiogram of a 3-year-old patient with an HHT mutation, following intracerebral artery (ICA) injection. Note the large left parietal AVM and small poster temporal AVM. Red caret = feeding arteries; blue caret = draining veins; asterisk = AVM. **D** Coronal (or anteroposterior) view. **E** Dorsal view of a P7 *Alk1*^*flox/flox*^ mouse following blue latex perfusion through the arterial circulation, shows normal formation of the arterial cerebrovasculature. **F** Same view of an *Alk1* induced endothelial cell knockout (*Alk1*^*iECKO*^) littermate, a model of HHT2, with obvious perfusion of the venous vasculature in the brain, indicative of arteriovenous shunts. The boxed in, magnified view shows a perfused artery (red caret) and a perfused vein (blue caret), which is not observed in control animals. The white asterisk denotes the vascular anomaly. BA = basilar artery; C.o.W. = Circle of Willis; br. st. = brain stem; MCA = midcerebral artery; ob = olfactory bulb

HHT arises from decreased bone morphogenetic protein 9/10 (BMP9/10) signaling (Fig. 2) [26, 27]. Briefly, in the presence of the ligands BMP9 or 10, the type III transforming growth factor beta (TGF-β) accessory receptor, Endoglin, facilitates binding of a heteroduplex of two constitutively active TGF-β type II receptor serine/threonine kinases (BMPR2 or ActRIIA are the main type II receptors expressed by ECs), which then interacts with two type I serine/threonine kinase receptors (in ECs, these are ALK1). Phosphorylated ALK1, in turn, phosphorylates the transcription factors SMAD1 and SMAD5, which facilitates their binding to SMAD4 [28–31]. This trimeric SMAD1/5-SMAD4 complex then translocates to the nucleus to regulate transcription of target genes (Fig. 2).

Loss of function mutations in either *ENG* (encoding Endoglin, ENG) or *ACVRL1* (encoding activin receptor-like kinase 1, ALK1), underlie the subtypes HHT1 and HHT2, respectively [31–34]. In 2013, Wooderchak-Donahue and colleagues identified heterozygous pathogenic missense variants in *GDF2* (chromosome 10q11.22 in humans, which encodes BMP9) in three patients with recurrent epistaxis and dermal telangiectasias (although with a slightly different distribution more similar to CM-AVM than classical HHT) [35]. Subsequent studies linked loss of function mutations in *GDF2* to HHT5, a rare form of HHT (a convention that will be maintained in this review) [36–39]. Loss of function mutations within *MADH4* (*Mothers against decapentaplegic homolog 4*, the gene encoding SMAD4), are responsible for the combined syndrome juvenile polyposis-HHT overlap (JP-HHT) and have also been implicated in bAVM formation [40]. Overall, causative mutations in either *GDF2*, *ENG*, *ALK1*, or *SMAD4* are found in 97% of patients with a definite clinical diagnosis of HHT [23].

BMP9/10 signaling promotes vascular maturation and endothelial quiescence [41]. Thus, disruption of this pathway upregulates vascular endothelial growth factor-A (VEGF-A) signaling and angiogenesis. We refer readers to the excellent review by Al Tobosh and colleagues for a more comprehensive discussion on HHT and BMP signaling [42]. Notably, multiple murine models of HHT have been created, providing valuable insights into bAVM pathogenesis, which we will describe below (and they are listed in Table 2).

**Table 2:**
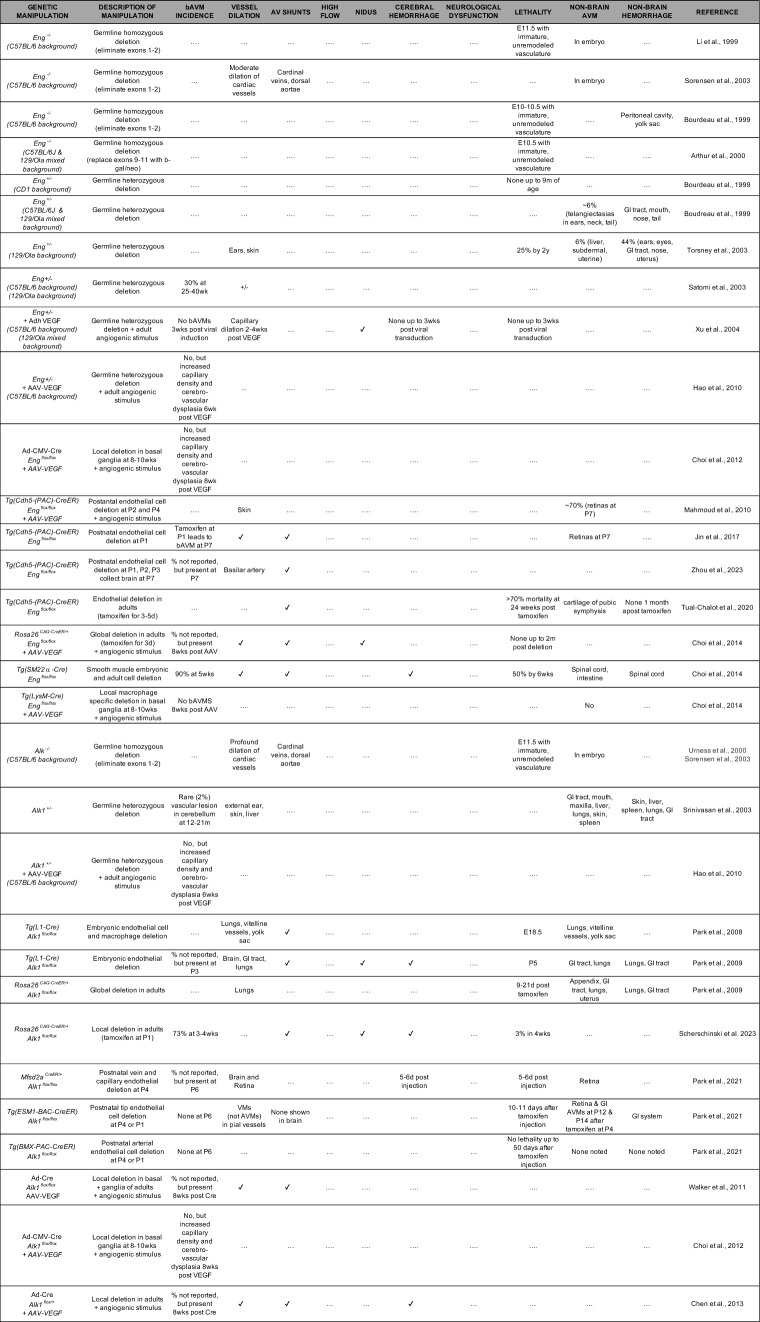

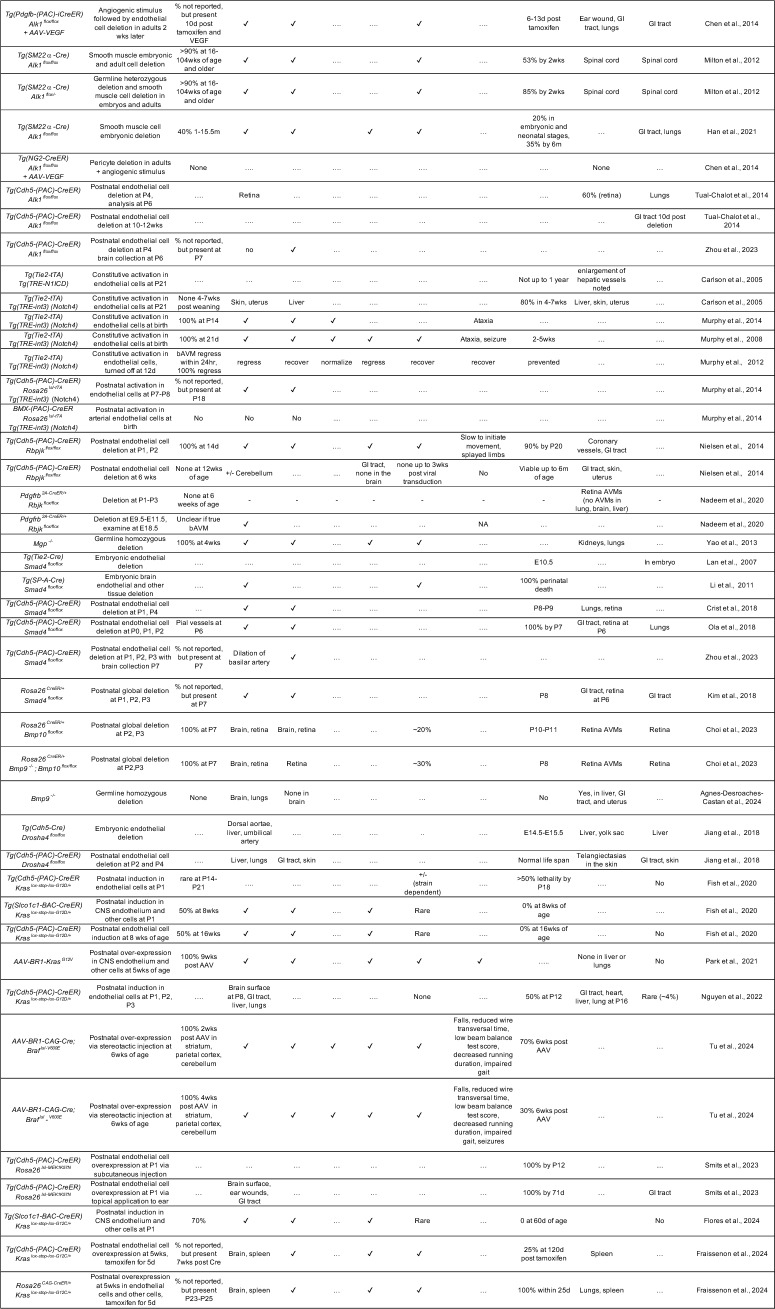
Mouse models of HHT and brain arteriovenous malformations

### Mouse models of HHT

While initial specification of the dorsal aortae and cardinal veins via vasculogenesis is intact in *Eng*^–/–^ mutant murine embryos, the yolk sac vasculature fails to remodel, and mutants exhibit cardiac cushion defects, decreased vascular smooth muscle cell recruitment, and delayed vessel maturation, all of which leads to mid-gestation embryonic lethality [43]. *Alk1*^–/–^ embryos display a similar phenotype, with defects in angiogenesis, smooth muscle cell recruitment, and embryonic lethality [44]. The presence of extensive arteriovenous shunts, and the downregulation of arterial markers, such as *Efnb2*, led to the hypothesis that *Alk1* loss disrupts early arteriovenous differentiation [44]. Subsequent analysis revealed that *Eng*^−/−^ embryos, similar to *Alk1* homozygous null mutants, possess discrete AVMs within the early axial vessels, further solidifying the link between ALK1, ENG, TGF-β and arteriovenous patterning [45]. However, unlike *Alk1* mutants, expression of arterial markers, such as *Efnb2*, is normal, vessels are minimally dilated, and AVM formation is delayed in *Eng*^*−/−*^ embryos [45].

One of the most surprising observations in these murine models was that, unlike human patients, defects such as bAVMs [46], vessel dilation [46–48], extracranial AVMs [49], and vessel hemorrhage [48, 49] are quite rare and generally mild in *Eng* or *Alk1* heterozygous mutants. bAVMs, specifically, are found in only a fraction of these animal models and do not present until late adolescence or early adulthood, if at all. The incomplete penetrance of these phenotypes, combined with the fact that the genetic background of the mice influences the frequency and severity of HHT-like defects, suggested heterozygous loss of TGF-β signaling components, while necessary, may not be sufficient to induce AVMs [46, 49–52]. However, both *Eng*^*−/*+^ and *Alk1*^*−/*+^ adult mice feature abnormal microvessel morphology (dilated, tortuous, etc.) and increased capillary density when administered a pro-angiogenic stimulus (i.e., VEGF-A), although bAVMs were not reported in these early studies [47, 53].

Additional murine models of *Alk* or *Eng* loss of function, through either tissue-specific conditional loss of function, or via focal deletion, have emerged for studying bAVMs in HHT. Induced, ubiquitous *Alk1* deletion in adult mice using a *ROSA26*-*CreER* driver line (*R26*^*CreER/*+^; *Alk1*^*flox/flox*^) led to death 2–3 weeks after administration of tamoxifen, precluding the long-term analysis of these animals [54]. While bAVMs were not reported in this initial study, stereotactic delivery of adenoviral Cre in conjunction with AAV-VEGF (VEGFA/VEGF_165_) in *Alk1*^*flox/flox*^ adult mice induced cerebrovascular abnormalities, including AV shunts and dysplastic vessels, reminiscent of bAVM [55]. Notably, another group reported that delivery of adenoviral Cre in conjunction with AAV-VEGF-A did not induce bAVMs in either adult *Alk1*^*flox/flox*^ or *Eng*^*flox/flox*^ mice, but did lead to cerebrovascular dysplasia (a caveat to these studies was the reportedly low transduction efficiency and even lower recombination rate for the *Eng* allele) [56].

Unlike the lethality observed shortly after induction of systemic *Alk1* deletion in adult mice, adult *Rosa26*^*CreER/*+^*; Eng*^*flox/flox*^ animals are viable for at least 2 months following tamoxifen induction [57], although another study showed that multiple high doses of tamoxifen led to lethality in adult *Rosa26*^*CreER/*+^*; Eng*^*flox/flox*^ mice [58]. However, like studies in *Alk1* mice, adult *Rosa26*^*CreER*^*; Eng*^*flox/flox*^ animals treated with tamoxifen did not present with bAVMs unless they were also subjected to stereotactic delivery of AAV-VEGF [57]. Notably, a robust, adult model of HHT-driven bAVM has emerged where stereotactic intracranial injection of tamoxifen in adult *Rosa26-CreER;Alk1*^*floxflox*^ mice leads to focal *Alk1* deletion in all cell types near the injection source and yielded localized and profound bAVMs, bypassing the lethality associated with systemic *Alk1* deletion in adult mice [59]. Whether a similar strategy will be effective in *Eng* mouse models remains to be determined.

The transition from the global deletion approaches, as well as the more recent localized multi-tissue loss of function models, to tissue-specific studies has shed light on the cellular requirements for HHT-related genes in the cerebrovasculature. Deletion of either *Eng* or *Alk1* in smooth muscle (as well as a fraction of the endothelium due to the use of the *SM22a-Cre* line) during embryogenesis yielded robust, progressive bAVM formation in postnatal animals without the need for a pro-angiogenic or -inflammatory cue [57, 60, 61]. However, approximately half of all *SM22a-Cre; Eng*^*flox/flox*^ mice die by 6 weeks of age [57]. *SM22a-Cre; Alk1*^*flox/flox*^ mice also exhibit neonatal lethality [61]. However, *SM22a-Cre* induces recombination in a small fraction of endothelial cells [61], leading some to hypothesize that the AVMs evident in these mice were due to the endothelial activity of *SM22a-Cre*, rather than a requirement for *Alk1* or *Eng* in smooth muscle [62]. In agreement with this model, smooth muscle-specific deletion of *Alk1* or *Eng* in adult mice using *Myh11-CreER* failed to induce AVMs in the skin (the brain was not reported) [58]. Pericyte-specific *Eng* deletion mediated by *NG2-CreER* also did not produce bAVMs in adult mice [62]. As mural cells exhibit extensive heterogeneity, with no single marker effectively labeling all pericyte subtypes [63], these murine studies should be viewed as a work in progress, rather than a definitive demonstration that pericyte-specific loss of HHT genes does not drive bAVMs.

Constitutive endothelial-specific deletion of *Alk1* via *Tg(Alk1-Cre)* is embryonic lethal in mice and results in vessel dilation [64] and frank hemorrhage in the brain, lungs, and gastrointestinal tract [54]. Early postnatal *Alk1* deletion using *Cdh5-PAC-CreER* (inducible endothelial-cell knockout, or *Alk1*^*iECKO*^) also rapidly leads to death [65], with arteriovenous shunts, dilated vessels, and other hallmarks of bAVM within the cerebral vasculature [66]. Notably, mutant *Alk1* ECs in distinct vascular beds may uniquely contribute to AVMs, as a recent study found that *Alk1* loss at P4 in CNS capillary and venous ECs (using *Mfsd2a*^*CreER/*+^), but not CNS arterial ECs (via *BMX-PAC-CreER*) led to retinal and brain AMVs by P6 [67]. *Mfsd2a*^*CreER*^; *Alk1*^*flox/flox*^ neonates died 5–6 days after tamoxifen injection, while survival was not impacted in *BMX-PAC-CreER*; *Alk1*^*flox/flox*^ mice up to 50 days following treatment. Of note, the evidence for brain AVMs in *Mfsd2a*^*CreER*^; *Alk1*^*flox/flox*^ animals was limited to a single image of a latex perfused brain with no quantification or higher resolution imaging shown. Intriguingly, *Alk1* deletion in tips cells and their derivatives using *ESM1-BAC-iCreER* led to vascular malformations in the retina and brain (but not true AVMs) and bona fide AVMs and hemorrhage in the gastrointestinal system, as well as death 10–11 days later [67].

*Eng* disruption in the early postnatal endothelium via *Cdh5-PAC-CreER* is also lethal within days and yields a similar phenotype in the brain, with evident bAVMs [66, 68]. Curiously, while endothelial-specific *Alk1* deletion in adult mice leads to death within 6–13 days following tamoxifen administration [62], endothelial-specific *Eng* loss in adult mice is initially well tolerated [58], but eventually leads to high output heart failure and roughly 70% mortality 24 weeks after tamoxifen treatment [69]. To date, bAVMs have not been reported in either adult endothelial-specific knockout model. However, stereotactic injection of AAV-VEGFA, followed two weeks later by endothelial cell-specific *Alk1* deletion using *Pdgfb-iCreER* [70], was sufficient to induce cerebrovascular dysplasia and malformed vessels reminiscent of bAVM in the adult mouse brain [62]. In agreement with the results of Park et al. [67] showing a requirement for *Alk1* in the capillary and venous endothelium of the retina and brain, postnatal *Eng* deletion in capillary and venous cells at P5 and P6 using *Apj-BAC-CreER* led to AVMs in the retina by P11 [71]. Unfortunately, this study did not remark on AVMs in other locations, such as the brain.

*GDF2* (BMP9) and *BMP10* each encode high-affinity ligands for ALK1 capable of inducing SMAD1/5 phosphorylation and expression of downstream target genes (i.e., *Id1*, etc.), and their expression overlaps in many tissues (with a few notable exceptions, such as the enrichment of *Bmp10* in the embryonic heart, and *Bmp9* in the early liver) [72–80]. These biochemical similarities and overlapping expression domains led some to speculate they are functionally redundant ligands for ALK1. However, while *Bmp9*^*−/−*^ mice do not have embryonic vascular phenotypes and survive to adulthood [81–83], *Bmp10*^*−/−*^ mice phenocopy *Acvrl1*^*−/−*^ in terms of embryonic lethality and the presence of AVMs [81]. Moreover, inserting *Bmp9* into the *Bmp10* locus (i.e., *Bmp10*^*Bmp9/Bmp9*^) rescues these embryonic AVMs [81], and only simultaneous blockade of BMP9 and 10 together (but not separately) leads to AVMs in the neonatal retina, suggesting these two ligands are functionally redundant [84–86]. In support of this model, there is a short window (E8.0-E9.75) when *Bmp10*, but not *Bmp9*, is expressed in the developing mouse embryo [87], potentially accounting for the embryonic lethality in *Bmp10*^−/−^, but not *Bmp9*^−/−^, mice. However, evidence from human genetics and zebrafish suggests *BMP10* may encode the dominant Alk1 ligand in ECs, and BMP9 cannot rescue cardiac defects in *Bmp10* mutants [81, 88]. Moreover, both *Rosa26*^*CreER/*+^; *Bmp10*^*flox/flox*^ and *Rosa26*^*CreER/*+^; *Bmp10*^*flox/flox*^; *Bmp9*^*−/−*^ mice feature AVMs in the postnatal retina and brain, as well as early neonatal lethality (unlike *Bmp9*^*−/−*^ mice) [82]. Administration of recombinant BMP10, but not BMP9, prevented retinal AVM in these *Rosa26*^*CreER/*+^; *Bmp10*^*flox/flox*^; *Bmp9*^*−/−*^ mice and moderately extended their survival [82]. This same study showed that global deletion of *Bmp10*, or combined loss of *Bmp9/10* in adult mice, led to death within 8 days, as well as wound-induced AVMs in the skin (bAVMs were not investigated) [82]. Of note, an independent study showed that systemic *Bmp10* deletion in 3 week old mice did not lead to lethality, despite targeting the same region of *Bmp10* (exon 2) and using the same CreER driver (*R26*^*CreER/*+^) and tamoxifen treatment regimen [89]. However, Bouvard et al. did report cardiomegaly, splenomegaly, and pulmonary hemosiderosis and indirect evidence of arteriovenous shunts or large dilations in the pulmonary vasculature of their Bmp9/10 double knockout animals [89]. In addition to these genetic models, administration of BMP9/10 blocking antibodies to neonatal mice also induces HHT-like phenotypes between P5 and P7, including GI and retinal AVMs [83, 85, 86]. Intriguingly, a recent study showed that germline loss of *Bmp9* (*Bmp9*^*−/−*^*)* led to enlarged vessels (but no AVMs) in the adult brain and lungs, with AVMs in the liver, gastrointestinal tract, and uterus [89]. Collectively, these studies indicate that loss of TGF-β (BMP9/10) signaling in the early, remodeling, angiogenic endothelium is a primary driver of bAVMs.

Until recently, little was known regarding the role of SMADs in the context of HHT. Constitutive endothelial-specific loss of *Smad4* is embryonic lethal [90], while constitutive brain endothelial-specific *Smad4* disruption resulted in intracranial hemorrhage and postnatal lethality [91]. Inducible systemic *Smad4* loss at P1-P3 via *Rosa26*^*CreER/*+^ leads to AVMs in the retina and brain (and other organs) and postnatal lethality by P8, similar to loss of either *Alk1* or *Eng* [92]. Induction of endothelial-specific *Smad4* loss at P1 is also lethal between P8-P9, and animals feature AVMs in the retina [93, 94] and brain [66]. Mechanistically, endothelial-specific *Smad4* loss altered endothelial cell shape and size within the retinal vasculature [93, 95]. In adult mice, global *Smad4* loss via *Rosa26*^*CreER*^ led to AVMs in the gastrointestinal tract and in the dermis following wounding, along with rapid lethality; however, bAVMs were not studied in these adult mice [92]. A recent study also showed that endothelial-specific loss of *Smad1* and *Smad5* (*Cdh5-PAC-CreER*; *Smad1*^*flox/flox*^; *Smad5*^*flox/flox*^) at P1 led to lethality by P6, as well as retinal AVMs with incomplete penetrance [96].

Despite the germline inheritance of these loss of function variants in HHT patients, bAVMs are diagnosed at various times throughout life [6]. Furthermore, while HHT is an autosomal dominant syndrome, in humans these malformations present as discrete, focal lesions while most other vessel beds remain unaffected [97]. These findings, and the fact that bAVMs are incompletely penetrant in *Eng*^+/–^ and *Alk1*^+/–^ mice, suggest reduced gene dosage alone is not sufficient to drive lesion formation [46, 49, 51, 52].

Instead, it has been proposed that development of an AVM requires not only a “first hit” (i.e., germline loss of function in one allele of either *ENG* or *ALK1*), but also an additional physiologic, environmental, or genetic “second hit” (e.g., somatic mutation of the remaining wild-type allele) [98, 99]. This model is consistent with findings in HHT patients, as the tissues most prone to developing telangiectasias are those subjected to repetitive injury, damage and repair: the face, lips, and fingers. Furthermore, recent human genetic studies suggest that a Knudsonian-esque two-hit mechanism explains the focal (rather than systemic) nature of vascular malformations in HHT patients, indicating that bi-allelic loss, rather than haploinsufficiency, underlies AVM pathogenesis [100, 101]. Given these findings, it appears that telangiectasias and AVMs in HHT may be more similar to sporadic bAVMs than initially anticipated, as they likely both arise from somatic, low frequency, mutations in the remaining normal allele leading to loss of heterozygosity, or through the acquisition of low frequency gain of function mutations in an oncogene (discussed below).

Studies in animal models further support this hypothesis, as AVMs develop more frequently in either *Alk1*^+/–^ or *Eng*^+/–^ adult mice in the presence of factors that stimulate inflammation and new vessel growth, such as physical wounding or the addition of exogenous VEGF-A (Table 1) [47, 53–58, 62, 102–104]. Moreover, endothelial-specific deletion of either *Alk1* (*Pdgfb-CreER*; *Scl-CreER*) or *Eng* (*Cdh5-PAC-CreER*; *Scl-CreER*) in adult mice is not sufficient to induce bAVMs without the addition of an angiogenic or inflammatory stimulus [58, 62, 104]. Relatedly, a follow up study showed that bAVM induction in *Scl-CreER*; *Eng*^*flox/flox*^ mice diminished as postnatal development progressed, such that induction of recombination between P22-P24 failed to induce vascular malformations in the brain [105]. Notably, this window directly coincided with VEGFR2 expression in the brain, which peaks during the initial 2 weeks following birth. The induction of bAVM induction during this developmental pro-angiogenic window, but not in later more quiescent stages, is also observed in NOTCH gain of function mouse models (discussed below). These findings, along with reports of high VEGF-A levels in bAVMs, further supports a two-hit hypothesis for lesion formation in HHT [106].

While most HHT patients feature damaging variants in genes encoding BMP/TGF-β signaling components, other forms of HHT have been identified [23]. Specifically, HHT3 involves two unidentified genes on chromosome 5, while HHT4 is associated with unidentified genes on chromosome 7 [107, 108]. Additionally, exome sequencing of individuals with two or more diagnostic criteria for HHT revealed an overrepresentation of missense variants in DROSHA (7.14% vs. 0.04%) [109]. Furthermore, a parent and child in a single family that both presented with multiple pulmonary AVMs and extensive epistaxis each possessed a pathogenic splice site mutation in *ENG* as well as an additional, potentially damaging missense variant in *DROSHA* [109].

*DROSHA* encodes an RNAse III endonuclease that, along with DGCR8 and the RNA helicase p68 (DDX5), forms a multiprotein microprocessor complex that mediates nuclear processing of primary microRNAs (pri-miRNAs) [110]. DROSHA activity is affected by BMP signaling activity, as BMP4 stimulation of the type I BMP receptor leads to phosphorylation and nuclear accumulation of p-Smad1/5/8. There, p-SMAD1/5/8 binds to p68, and together with DROSHA and DGCR8, promotes the accumulation of a subset of pre-miRNAs that go on to form functional microRNAs, so called BMP-Smad-dependent miRNAs (Fig. 2) [109, 111]. Critically, binding of the DROSHA missense variants identified in patients to p-SMAD1/5/8 and p68 was decreased compared to wildtype DROSHA, suggesting these mutations reduced processing and generation of BMP-SMAD-dependent microRNAs.

Endothelial specific deletion of *Drosha* (*e*ndothelial *c*ell-specific *k*nock*o*ut, or *Drosha*^ECKO^) is embryonically lethal between E14.5-E15.5, and mouse embryos present with disorganized liver vasculature, dorsal aorta dilation, and a torturous extraembryonic vasculature [109]. Inducible endothelial-specific *Drosha* deletion (*Cdh5*-*CreERT2; Drosha*^*flox/flox*^,* or* *i*nducible *e*ndothelial *c*ell-specific *k*nock*o*ut/*Drosha*^iECKO^) at P2 and P4 did not affect survival, or lead to AVMs in the lungs or livers, but did lead to misaligned vessels in the GI tract, as well as intestinal hemorrhage, and AV fistulas in the cutaneous vasculature [109]. AVMs within the brain were not reported. Despite these vascular defects, and their partial phenotypic overlap with *Eng* and *Alk1* mutants, AVMs with a defined, remodeled nidus were not shown in either *Drosha*^ECKO^ or *Drosha*^iECKO^ mutants [109]. While these data suggest DROSHA dysfunction may play a role in HHT, the exact targets of these BMP-miRNAs in AVMs remain to be determined.

### Altered Notch signaling drives bAVMs in mice

**Fig. 3:**
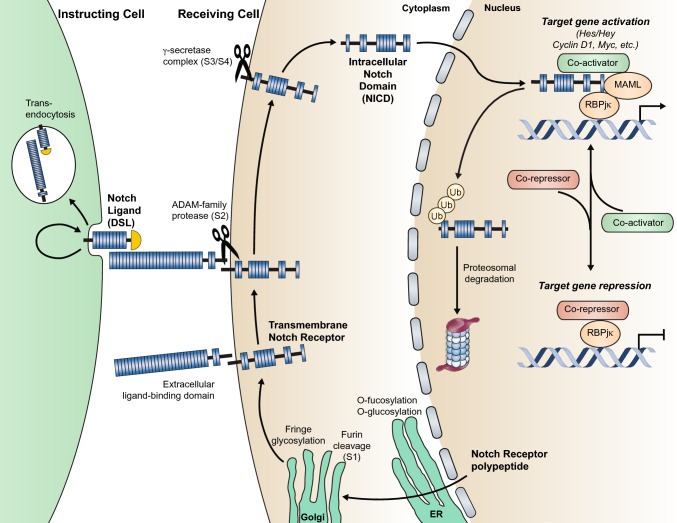
The Notch signaling pathway. The Notch receptor is transcribed and translated and the polypeptide exported from the cell, where serine and threonine residues within the epidermal growth factor (EGF) repeats of the extracellular domain are O-fucosylated and/or O-glucosylated by the enzymes Pofut and Poglut, respectively, two essential modifications for generating a fully functional receptor. The receptor is further modified by the glycosyltransferase Fringe, as well as xylotransferases, and this “sugar code” on the EGF repeats of the extracellular domain of Notch alters the affinity of the receptor for different ligands. While in the golgi, the polypeptide is proteolytically cleaved by Furin (S1). The cleaved heterodimer is assembled and held together via non-covalent interactions and trafficked to the cell surface. Binding to one of the Delta, Serrate, or Jagged (DSL) ligands on an adjacent cell leads to receptor activation via juxtacrine signaling. Briefly, ligand tension and subsequent endocytosis generate the force necessary to induce a conformational change in the bound receptor that exposes site 2 (S2) for cleavage by ADAM metalloproteases. This cleavage generates the membrane anchored Notch extracellular truncation (NEXT) fragment, a substrate for the γ-secretase complex. γ-secretase cleavage at site 3 (S3) and (S4) results in release of the Nβ peptide and the Notch intracellular domain (NICD). In the absence of Notch signaling, RBPjκ (CBF1/CSL/Su(H)/Lag-1) associates with corepressor proteins and histone deacetylases to repress transcription of target genes. Following ligand activation and receptor cleavage, the NICD translocates to the nucleus and binds to RBPjκ to displace the repressors and recruit the coactivator Mastermind (MAML), which in turn recruits additional coactivators to stimulate transcription. Adapted from Kopan and Ilagan, 2009 [112]

The Notch family (*NOTCH1-4* in mice and humans) encodes large, single-pass type I transmembrane receptors. Following ligand binding, the receptor is cleaved extracellularly by ADAM proteases, then intracellularly by γ-secretase, which allows the Notch intracellular domain (NICD) to translocate to the nucleus and interact with its binding partner, the transcription factor Recombination signaling-Binding Protein for immunoglobulin-k J (RBPjκ). NICD binding to RBPjκ on DNA induces recruitment of transcriptional co-factors and activators, allowing induction of Notch target genes (Fig. 3).

While germline or somatic variants within the NOTCH signaling pathway in bAVM patient samples have not been reported, an association between SNPs in the region coding for the extracellular epidermal growth factor-like domains (the ligand binding region) of NOTCH4 has been associated with AVM development and hemorrhage [113]. Moreover, expression of Notch receptor ligands, including *JAG1* and *DLL4*, as well as the downstream Notch effector *HES1*, are increased in human bAVMs [114]. However, reports offer conflicting findings, as the aforementioned study documented increased *NOTCH1* expression in the smooth muscle and endothelium of human bAVMs [114]. In contrast, immunohistochemical screening of tissue microarrays from surgically resected bAVMs and control tissue from epilepsy brains revealed NOTCH 1 was decreased in bAVM tissue (while expression of both NOTCH3 and 4 was elevated) [115]. The authors of this same survey remarked that immunohistochemistry failed to detect NOTCH2 in either control or bAVM tissue [115].

Early arteriovenous fate in vertebrates requires normal Notch signaling, as loss of Notch1, its ligand Dll4, or the transcriptional effector Rbpjk, as well as the downstream transcriptional targets Hey1 and Hey2, lead to arteriovenous patterning defects in fish and mice [116–122]. For a comprehensive discussion on the role of this pathway in early vasculogenesis, we refer readers to Fish and Wythe, 2015 [123]. Given this essential role in establishing early embryonic arteriovenous identity, as well as regulating developmental and pathological angiogenesis, multiple studies have focused on the postnatal role of canonical Notch signaling in murine arteriovenous identity and patterning. Endothelial-specific, tetracyline (tet)-inducible expression of a dominant active Notch4 intracellular domain (N4ICD) in adult mice (*Tie2-tTA*; *TRE-N4ICD*) led to evident AVMs within multiple tissues (liver, skin, and uterus), but not the brain [124]. However, early postnatal induction of *Notch4* expression led to bAVMs by 3 weeks of age and death by 5 weeks, with increased expression of arterial markers (*Efnb2*, *Cxn40*, *Jag1*, *Dll4*) and reduced venous gene (*EphB4*) expression [125, 126]. Thus, like HHT and TGF-β loss of function models, Notch gain of function in neonatal, but not adult mice, results in bAVMs. Expression of a dominant active Notch1 intracellular domain (N1ICD) was also sufficient to induce bAVMs in neonates but not adult mice [127]. Critically, Nielsen and colleagues demonstrated that *Notch4*-driven bAVMs in mice require the canonical Notch transcriptional co-factor Rbpj (also known as CSL or CBF), as endothelial-specific *Rbpj* loss suppressed tet-inducible, endothelial-specific Notch4 gain of function-driven bAVMs in postnatal mice [128].

Mechanistically, these vascular anomalies appear to arise from microvessels, as Notch gain of function increases the diameter of capillary-like vessels without affecting EC proliferation [127]. Remarkably, these lesions require constant Notch signaling, as silencing of the doxycycline-regulated transgene reversed pathology and normalized vessel architecture [124, 126]. Curiously, *Rbpjk* deletion in the postnatal endothelium, and thus loss of Notch activity, also leads to features of bAVM in mice by P14, suggesting that too much or too little Notch signaling can contribute to bAVMs [129, 130]. While *Rbpjk* deletion from P10-P13 yielded larger vessels within the retina by P28 due to endothelial hyperplasia, arterial-specific loss of *Rbpjk* using *Bmx-PAC-CreER* (following a similar time course) failed to alter arterial patterning or smooth muscle cell recruitment, suggesting canonical Notch signaling is dispensable in the late postnatal arterial endothelium [131]. *Pdgfb-BAC-CreER* driven (endothelial-specific) inactivation of the gene encoding the NOTCH1 and NOTCH4 ligand, *Dll4*, led to defective arterial patterning in the retina, similar to NOTCH1 intracellular domain (N1ICD) overexpression, although the presence or absence of AVMs was not discussed in that manuscript [131]. Interestingly, bAVMs often feature reduced pericyte coverage [132], and recent work demonstrates that disruption of Notch signaling via ablation of Rbpj in these supporting mural cells (*Pdgfrβ*^*2A−CreERT2/*+^*; Rbpjκ*^*flox/flox*^) at P1-P3 led to AVMs in the retina at P14. Expression of a dominant negative MAML—a Notch transcriptional co-activator—in pericytes (*Pdgfrβ*^*2A−CreERT2/*+^*; R26*^*lsl−dnMAML*^) confirmed these results. Interestingly, *Rbpjk* deletion in vascular smooth muscle cells (via *Act2a-CreER*) did not induce AVMs in the retina, suggesting the importance of these capillary EC-pericyte interactions. Postnatal *Rbpjκ* deletion at P1-P3 in pericytes also did not lead to bAVMs in adult mice, although tamoxifen delivery from E9.5-E11.5 did yield vascular malformations in the murine forebrain at E18.5 (it is unclear if these anomalies were genuine bAVMs). Overall, these results confirm the importance of Notch signaling in pericytes in AVM (and potentially bAVM).

Interestingly, crosstalk between Notch and other signaling pathways, such as TGF-β, may play a role in AVM pathogenesis. For instance, recent studies show ALK1-dependent SMAD signaling induces expression of the downstream Notch targets HEY1 and HEY2, which in turn represses VEGF-A signaling and limits angiogenic sprouting [133]. Furthermore, combined blockade of NOTCH and ALK exacerbates *Alk1*-dependent phenotypes in vivo, including angiogenic hypersprouting. Conversely, BMP9 activation of Alk1 rescues Notch loss of function defects [133]. Additionally, germline homozygous deletion of *matrix Gla protein* (which encodes a BMP inhibitor) also led to bAVMs by 4 weeks via activation of ALK1, a BMP receptor, which was associated with increased Notch activity [134]. Thus, *ALK1-*driven defects may involve dysregulated Notch signaling. Although Notch plays a fundamental role in arteriovenous differentiation and endothelial identity, much remains to be determined regarding the translation of these findings to bAVMs and human disease.

## The genetic basis of sporadic bAVMs

### Somatic activating KRAS mutations drive sporadic, de novo bAVMs

Despite the critical gains made in understanding the genetic causes and mechanisms in inherited diseases featuring AVMs, until recently we lacked a fundamental understanding of the etiology underlying the most frequent type of bAVMs: sporadic bAVM. This changed in 2018, when we discovered mutations within one gene, *KRAS,* in more than 50% (45 of 72 samples) of sporadic bAVM samples from a patient cohort collected in Toronto, Canada and in a validation cohort from Kuopio, Finland [135]. Importantly, these patients presented with a unifocal lesion (i.e., one bAVM per patient) without any familial history of AVMs or inherited vascular disease.

KRAS, like all members of the RAS family of small GTPases, functions as a binary switch, cycling between an inactive guanosine diphosphate (GDP)-bound state and an active guanosine triphosphate (GTP)-bound state. Guanine exchange factors (GEFs) alter the affinity of RAS for GDP, leading to its exchange for GTP and its activation, while GTPase-activating proteins (GAPs) promote GTP hydrolysis, acting as negative regulators of RAS and down-regulating activity of their targets. GEF and GAP regulation of RAS depends upon their recruitment to the plasma membrane and their physical association with the GTPase. Once activated, RAS induces numerous downstream effectors (including the PI3K/AKT signaling pathway and the RAF-MEK-ERK kinase cascade [136]) to induce gene expression, cell proliferation, survival, and differentiation [137].

The somatic mutations we identified were confined to ECs and resulted in missense variants predominantly in glycine 12 and rarely in glutamine 61. Specifically, in the discovery and validation cohorts, 31/72 patients had c.35G → A (p.Gly12Asp) substitutions, while 13/72 had c.35G → T (p.Gly12Val) and 1/72 had c.183A → T (p.Gln61His). These missense mutations of glycine 12 prevent GAP-mediated hydrolysis, leading to accumulation of GTP-bound RAS. Similarly, replacement of glutamine 61 impairs both RAS intrinsic GTP hydrolysis and GAP-mediated nucleotide exchange. The frequency of activating variants in *KRAS*, as well as the predominance of G12D and G12V variants compared to Q61H, and the identification of rare G12C mutations, has since been validated in numerous additional patient cohorts [138–143]. Other mutations in *KRAS*, a potent oncogene in non-endothelial cells, increase nucleotide exchange and decrease GAP-dependent GTP hydrolysis (i.e., G13 variants), or do not alter GTP hydrolysis but instead increase intrinsic and GEF-mediated exchange of GDP for GTP (i.e., K117), or reduce overall affinity for GDP (i.e., A146 mutations) [144, 145]. However, to date only variants in G12 and Q61 have been described in bAVMs.

Multiple studies, including histological analysis of bAVM sample tissues [135], western blot of endothelial cells cultured from patient lesions [135], and MEK-inhibition of endothelial cells overexpressing mutant KRAS [146], suggest that KRAS preferentially activates the RAF-MEK-ERK (or mitogen activated protein kinase, MAPK) pathway in the endothelium [147, 148], making this pathway an important focus of future research and targeted therapies. Curiously, mutations in other RAS family members, such as NRAS and HRAS, have not been identified in bAVMs.

Following the association of *KRAS* variants with bAVM, multiple studies identified *BRAF* variants in human bAVM tissue, although these mutations are less prevalent than *KRAS* (7.5% vs 55%, respectively) [138]. In 2019, analysis of 21 surgically resected bAVM and 10 spinal arteriovenous malformations (sAVM) identified a *BRAF* c.1799 T > A (p.V600E) mutation in one bAVM (4.8%) and one sAVM (10%) [140]. A distinct study of 22 brain AVM specimens identified *BRAF* p.V600E in one specimen (4.5%), as well as *BRAF* p.Q636X in another patient [139]. These studies suggest constitutive BRAF activity may drive bAVM pathogenesis and could also represent a potential therapeutic target in bAVM. Since KRAS and BRAF act in the same pathway, this further implicates RAS/MAPK signaling in bAVM pathogenesis. Currently, only variants in *BRAF*—not the other RAF serine/threonine kinase family paralogs, *ARAF* or *CRAF* (*RAF1*)—have been described in bAVM patients. Importantly, BRAF has the highest affinity for KRAS of the RAF kinase family, and it is the most potent activator of MEK1 (mitogen activated protein kinase/ERK kinase, encoded by *MAP2K1*), as well as the ERK pathway in vitro [149–154].

Somatic *MAP2K1* gain of function mutations are classically associated with extracranial AVMs [139, 155, 156], but at this time they have not been reported in bAVMs. Germline mutations in *MAP2K1* (which encodes MEK1) and *MAP2K2* (MEK2) can cause cardio-facio-cutaneous (CFC) syndrome, a known RASopathy, although there are no reports of bAVMs in these patients [155, 157–160].

The rapid pace of these genetic discoveries linking RAS-MAPK signaling to bAVM has been matched by studies in murine models, demonstrating a role for this signaling cascade as a causative driver of sporadic bAVM.

### Animal models of RAS/MAPK pathway-driven bAVM

**Fig. 4:**
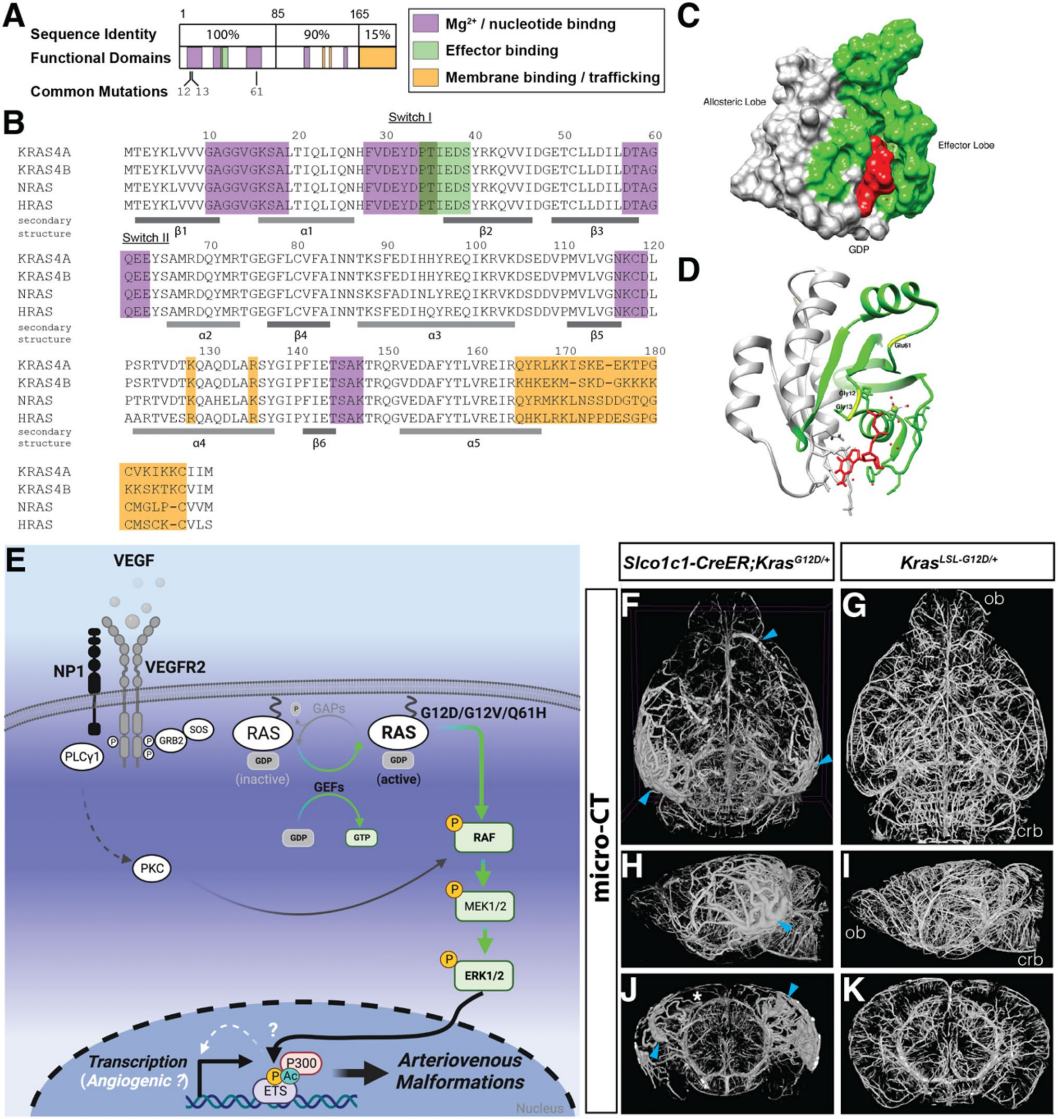
Mutant KRAS and Brain Arteriovenous Malformations. **A** The sequence identity and conservation of key functional domains across NRAS, HRAS, KRAS4A, and KRAS4B is shown. Somatic mutations described in the literature within KRAS are indicated at the bottom. Similar to known oncogenic mutations in RAS, they occur at codons 12, 13, and 61 and lead to constitutive GTPase activity. **B** Sequence similarity between the amino terminus of the four RAS isoforms, with protein domains highlighted. Mutations within and around loops 1, 2, and 4 (in Gly12, Gly13, and Glu61) affect nucleotide binding, resulting in enhanced GTP binding by RAS. **C** Overview of the 3D structure of RAS with the allosteric lobe shown in grey, the effector lobe highlighted in green, and GDP shown in red. **D** A structural view shows how these residues (Gly12, Gly13, Glu61) within the switch I domain (effector lobe) interact with GDP and GTP. Mutation within these residues prevents GTP hydrolysis, locking RAS in a constitutively active state. **E** Diagram of a receptor tyrosine kinase pathway, specifically VEGF-VEGFR2 signaling, that acts upstream of KRAS in the endothelium, and the molecular consequence of constitutive KRAS activity favors RAS-MAPK signaling in these cells. **F–K** Computed micro tomography (micro-CT) imaging of the cerebrovasculature of CNS-endothelial specific *Kras*^*G12D*^ mutant mice at 2 months of age following perfusion with a contrast agent reveals striking arteriovenous connections, or arteriovenous shunts, with a dilated feeding artery and draining veins (blue caret) as well as reduced small vessels in the cortex (asterisk). From Suarez et al. 2024 [163]. **F, G** Dorsal view: **H, I** sagittal view; **J, K** coronal view. Panels A and B are adapted from Prior IA et al. 2012 [164]

In 2020 our team was the first to model EC-specific somatic *KRAS* variants in vivo. By using a conditional, Cre-activatable, mutant *Kras*^*G12D*^ mouse strain (referred to as *Kras*^*lox−stop−lox−G12D*^ or *Kras*^*G12D*^) we demonstrated that post-natal, endothelial-specific expression of mutant *KRAS* in the endothelium was sufficient to induce bAVMs (Fig. 4) [146]. Importantly, in this model a *loxP* flanked stop cassette is located downstream of the endogenous Kras promoter, preventing transcription of a downstream exon containing the mutated G12D codon (*Kras*^*lox−stop−lox−G12D*^) [161]. Thus, while excision of the stop cassette relies on the endothelial-specific Cre driver, actual expression of mutant Kras is driven by the native promoter at physiologic levels (unlike, for instance, CAGGS driven over-expression in an *AAV-CAG-KRAS*^*G12D*^ transduced animal or in a *Rosa26*^*CAG−lsl−KrasG12D*^ mouse model). Induction of mutant *Kras*^*G12D*^ expression at postnatal day 1 (P1) with a pan-endothelial-specific, inducible Cre recombinase, *Cdh5-PAC-CreERT2*, led to intracranial hemorrhage and lethality beginning at P12, with ~ 50% dead by P18 [146, 162]. Notably, we failed to detect bAVMs at P21, as vessels appeared thin and fragile, rather than dilated and tortuous (nor could we explain the cause of mortality, as cardiac function was for the most part normal) (Table 2) [146].

The early postnatal lethality in the *Cdh5-PAC-CreERT2* line was bypassed by using a S*lc1o1c1-BAC-CreER* driver [165] to restrict *Kras*^*G12D*^ expression to CNS endothelial cells [146, 165]. This line offered another advantage, as it more accurately models the somatic acquisition of *KRAS* mutations in the brain in human disease. Examination of the cerebrovasculature at 8 weeks of age revealed an absence of intracranial hemorrhage, and fluorescence imaging showed that roughly 50% of all animals exhibited bAVMs containing dilated and tortuous vessels (Fig. 4). We also determined that induction of mutant *Kras*^*G12D*^ in adult mice using the pan-endothelial *Cdh5-CreER* driver did not affect survival or induce hemorrhage, but did lead to bAVMs in approximately 50% of all animals [146]. Another group showed that five-week-old mice virally transduced to overexpress KRAS^G12V^ displayed bAVM within three weeks (at 8 weeks of age), further supporting the idea that KRAS mutations promote bAVM development [166]. Despite the relative infrequency of G12C mutations in bAVM [167], our group found that *Slco1c1-BAC-CreER* driven recombination of a *Kras*^*lox−stop−lox−G12C*^ allele led to obvious bAVM in mice, in ratios similar to G12D mice (although some differences were evident in terms of location of the lesions in the brain) [163]. Fraissenon and colleagues also reported that tamoxifen induction at 5 weeks of age in *Cdh5-PAC-CreER*; *Kras*^*lox−stop−lox−G12C*^ led to AVMs in the brain and other organs within 7 weeks, with 25% lethality by 120 days post tamoxifen induction [167]. They also showed that systemic induction of Kras^G12C^ at 5 weeks of age in a *Rosa26-CreER*; *Kras*^*lox−stop−lox−G12C*^ model led to lethality within 30 days, with evident hemorrhage and AVMs throughout the animal [167].

Intriguingly, transgenic mice driving expression of mutant HRAS in the postnatal endothelium featured dilation of capillary vessels and cranial hemorrhage, but AVMs were not noted [168]. Relatedly, while loss of function mutations in negative regulators of RAS signaling (e.g., RASA1/p120 RAS-GAP) are associated with CM-AVM1 (capillary malformation-AVM) in humans, bAVMs are not a prominent feature in these patients [169, 170]. Similarly, while murine models of CM-AVM1 (*Rasa1* loss) and CM-AVM2 (*EphB4* loss) models present with vascular and lymphatic defects, they also lack bAVMs [171–175], suggesting a unique role for KRAS in bAVM pathogenesis.

Importantly, postnatal activation of the downstream KRAS effector, BRAF, variants of which have also been identified in bAVM (described above), also generates bAVMs in mice. Tu and colleagues delivered Cre via intravenous injection of *AAV-BR1-CAG-Cre-WPRE-*SV40pA—which displays selective tropism for the CNS endothelium [176]—to a novel *Braf* gain of function mouse line (which is not completely described in that manuscript, but it is implied that expression of a constitutively active BRAF^V600E^ variant depends upon Cre activity).This resulted in significant lethality within three to six weeks after transduction in adult mice [177]. However, focal delivery of *AAV-BR1-CAG-Cre-WPRE-pA* via stereotactic injection in the brain was well tolerated, and bAVMs formed in 100% (n = 7) of injected mice by 4 weeks post-injection, with a moderate rate of death, along with significant neurocognitive deficits and an inflammatory microenvironment [177]. One complication to interpreting these results is the established off-target (i.e., non-endothelial) activity of *AAV-BR1*, which also transduces both neurons and astrocytes [163, 176, 178–180]. This caveat is notable given the seizures, neuroinflammation, and death in *AAV-BR1-CAG-Cre* transduced *Braf*^*V600E*^ mice, as mutant BRAF expression in neurons leads to epilepsy and an inflammatory immune response, while expression in glial lineages (such as astrocytes) induces tumors, neuroinflammation, and death [181, 182]. Thus, given the off-target effects of *AAV-BR1*, whether endothelial-specific BRAF^V600E^ is sufficient to induce these phenotypes remains unclear.

Mutations in another RAF family member, *CRAF*, have not been associated with AVM or bAVM. However, endothelial-specific over-expression of CRAF^S259A^ (which cannot be phosphorylated by AKT, and is thus constitutively recruited to the plasma membrane for activation by RAS) alters arteriovenous patterning in the murine yolk sac and leads to embryonic lethality by E15.5 due to lymphatic vessel defects. Notably, overall arteriovenous patterning is spared in the embryo proper, and AVMs were not reported [183, 184].

Whether variants in *ARAF*, which have been associated with lymphatic vascular anomalies [185, 186], play a role in bAVM remains to be seen, although no reports have identified activating variants in this allele in bAVM at present.

A dominant active variant of the downstream MAPK kinase, MEK1^K57N^ (encoded by *MAPK2K1*)*,* led to sustained ERK1/2 phosphorylation in cultured endothelial cells, while constitutive expression in the murine embryonic endothelium in *Cdh5-Cre*; *Rosa26*^*lox−stop−lox−MAPK2K1−K57N−GFP*^ mice led to lethality at E16.5, with hemorrhage and vascular dysplasia in the skin, liver and brain evident at E15.5 [187]. Postnatal induction of MEK1^K57N^ expression at P1 in *Cdh5-PAC-CreER*; *Rosa26*^*lox−stop−lox−MAPK2K1−K57N−GFP*^ mice induced death between P10 and P12, similar to the lethality observed following tamoxifen injection at P1 in *Cdh5-PAC-CreER; Kras*^*lox−stop−lox−G12D*^ mice [146]. Notably, topical application of lower doses of tamoxifen in these endothelial-specific *Map2k1*^*K57N*^ mice led to death between P16 and P71, with vascular anomalies evident on the surface of the brain and skin; however, no bona fide arteriovenous shunts or niduses were shown in the brains of these mice in either report [156, 187]. Of note, the *Map2k1* transgene was not driven by the native *Map2k1* promoter, but by a strong synthetic CAG promoter at the *Rosa26* locus. Additionally, the authors propose that unlike RAS and RAF, mutant MAP2K1 will not elicit a negative feedback loop, although the reasoning for this is unclear given the role of both DUSP and SPROUTY proteins and their repression of MEK-ERK1/2 signaling [188].

Taken together, tremendous progress has been made in a relative short period of time (since 2018) in developing mouse models of the major genetic drivers of sporadic bAVMs. However, much work remains to be done in terms of characterizing the impacts of mutant RAS-MAPK activity at the organismal, tissue, and cellular levels in bAVM, both in the endothelium, as well as the surrounding neurovascular unit. Additionally, as these mutations often occur at a low variant allele frequency, determining the cell autonomous and non-autonomous impacts of these mutant ECs represents an important goal for the field. By incorporating novel methods for selective transduction in the CNS endothelium, such as *AAV9-BI30* or via CNS-specific transgenic Cre mouse lines, and leveraging existing mouse models pioneered for studies of oncogenes (such as *Kras*, *Braf*, and Map2k1 gain of function lines), the field will undoubtedly tackle these important questions. Much remains to be determined regarding the mechanisms of bAVM formation, maintenance and treatment, and these mouse models (as well as newly developed lines) will be crucial to the next wave of discovery.

## Molecular therapeutics: past, present, and future

The ongoing characterization of the mechanisms underlying bAVMs in HHT, and recent elucidation of molecular pathway alterations leading to sporadic bAVMs, have opened the door to novel pharmaceutical interventions and offers new hope to patients. Preclinical mouse models of bAVM have played a key role in demonstrating the potential and mechanistic basis of these novel treatments. Below we briefly describe promising therapeutic approaches that could be used in the future as possible front-line treatments.

### Thalidomide

Currently, there are no FDA approved drugs for treatment of HHT and bAVM; however, animal studies and clinical trials have identified several potential drugs targeting known pathways in bAVM development. One such drug, thalidomide, and its less toxic counterpart lenalidomide, have been shown to reduce nose bleeds and GI hemorrhage in HHT patients by inhibiting angiogenic factors like TGF-β and VEGF [189–191]. While these drugs have been previously used for symptomatic treatment of telangiectasias in HHT, new data suggests that these drugs can stabilize bAVM vessels in a mouse model, and, as a result, decrease bAVM hemorrhage. Zhu and colleagues found that thalidomide-treated *Alk1* mutant mice exhibited fewer dysplastic vessels and increased vessel coverage with vascular smooth muscle cells [192]. Treated mice also exhibited fewer hemorrhagic events and an overall increase in Pdgfb expression [192]. Overexpression of Pdgfb in mouse models was also found to reduce hemorrhage and dysplastic vessels, suggesting that thalidomide may work in part by upregulating Pdgfb. A recent case report study examining the effect of thalidomide in 18 patients with severe symptomatic peripheral AVMs (not bAVMs) found that all participants experienced rapid reduction in pain, cessation of bleeding, and ulcer healing [193]. Eight AVMs appeared stable after 58 months, one appeared cured, and four reoccurred after 11.5 months. The remaining five patients underwent embolization and were able to reduce their thalidomide dosage. While thalidomide may prove promising for treatment, it should be noted that this drug is associated with several side effects and is a known teratogen [194]. Accordingly, long-term administration must be carefully balanced against the potential benefit it offers. Second generation thalidomide analogues with a reduced toxicity profile, such as pomalidomide, do not yet have a long enough history of administration to secure full clinical endorsement. However, there is a multi-institutional open clinical trial of pomalidomide for reduction of epistaxis in HHT patients with promising early results (NCT03910244).

### Humanized anti-VEGF-A antibodies

Another potential medication for treatment of bAVM is Bevacizumab (Avastin), a humanized monoclonal antibody that blocks VEGF-A activity. Bevacizumab is used in HHT patients with severe GI mucosal bleeding and epistaxis, high output cardiac failure, hepatic vascular lesions, or severe hemorrhage, with studies showing marked clinical improvement in patients [195–198]. Limitations for its use include side effects such as hypertension, impaired wound healing and vascular pathology, joint pain, headache, and proteinuria [194, 196–198]. Like thalidomide, studies have also begun investigating bevacizumab as a treatment for bAVMs. Walker and colleagues demonstrated that bevacizumab treatment of conditional adult *Alk* mutant adult mice (Adenovirus-driven Cre; *Alk1*^*flox/flox*^) injected with AAV-VEGF-A (to induce bAVM) reduced both vessel density and the dysplasia index of bAVMs [199]. While promising in mice, few human studies have been performed. A recent pilot study on two patients with unresectable bAVMs revealed no change in lesion size over the course of 52 weeks when treated with bevacizumab. Patients reported no adverse events while on the medication [200]. Larger clinical studies are still needed to determine efficacy and safety. As bevacizumab treatment carries the potential side effect of bleeding (including intracerebral hemorrhage), its use in bAVM patients with a high chance of intracranial rupture carries significant risk and should likely be approached with caution.

### RTK inhibitors

Previous reports have shown that FDA approved oral receptor tyrosine kinase (RTK) inhibitors that target VEGFR2, such as pazopanib (Votrient), are beneficial in reducing intestinal bleeding, but not wound-induced AVMs in the skin in adult-induced *Alk1* mutant mice (*R26*^*CreER/*+^, *Alk1*^*flox/flox*^) [201]. Notably, pazopanib reduced epistaxis and increased hemoglobin levels in HHT patients in a multicenter proof of concept trial [202] and is currently under clinical efficacy study for HHT related epistaxis and anemia (NCT03850964). Whether this class of drugs can ameliorate bAVMs is unclear, as one may expect that patients harboring activating mutations within an effector downstream of VEGF or VEGFR2, such as *KRAS*, would see little benefit with this therapy. However, if mutant KRAS potentiates VEGF/VEGFR2 signaling through a positive feedback loop, or non-autonomously activates the pathway in neighboring cells, then this strategy may prove effective. Indeed, we previously observed that *VEGFA* and *VEGFC* transcripts were upregulated in cultured ECs over-expressing activated KRAS [135].

While RTK inhibitors traditionally suffer from a lack of target specificity (i.e., they may block VEGFR2 signaling, but they also disrupt TGF-β, FGF, EGF, PDGF and other RTK pathways), Nintedanib is of potential interest, due to its reported narrower spectrum of inhibition and minimal side effects. Indeed, a report showed that nintedanib, which is approved for treatment of idiopathic pulmonary fibrosis, reduced epistaxis and skin lesions in an HHT patient [203]. Notably, combinatorial administration of nintedanib and sirolimus (an mTOR inhibitor) rescued the vascular pathology of HHT in both genetic (*Alk* mutant) and drug-induced (BMP9/10ib treated) mouse models of HHT [204].

### PI3K/mTOR inhibitors

PI3 Kinase/mTOR signaling, a critical downstream effector in both the VEGF/VEGFR2 and ANGPT2/TIE2 pathways, has also been explored as a therapeutic target for bAVMs. Recently, reports showed that Tacrolimus, an inhibitor of mTOR, was associated with increased Smad1/5/8 signaling in cultured *Alk1* knock-down endothelial cells and in an *Alk1*^*−/*+^ mouse model of HHT and pulmonary arterial hypertension [205, 206]. These same studies showed that a macroside analogue of Tacrolimus, sirolimus (rapamycin), also activates Smad1/5/8 [206]. Thus, the immunosuppressant rapamycin prevents PI3K activation of mTOR, while simultaneously increasing Smad signaling [26]. Notably, genetic or pharmacologic disruption of PI3K also rescues defects in *Alk1* heterozygous mutants [85, 207]. Similar results for mTOR inhibitors were reported in an anti-BMP9/BMP10 blocking antibody (BMP9/10 immunoblocked or BMP9/10ib mice) model of HHT. Sirolimus has also shown efficacy as a treatment for blue rubber bleb nevus syndrome, a disease characterized by venous malformations in the skin and GI tract, as well as other lymphaticovenous malformations [208, 209]. Currently, sirolimus is the current standard of care and frontline therapy for many slow-flow vascular anomalies (lymphatic and venous malformations) [210]. However, while effective for these lesions, very few reports show success in managing high-flow AVMs. Thus, whether sirolimus (mTOR inhibitor) or Alpelisib (PIK3CA inhibitor) will become frontline treatments for bAVMs and fast-flow vascular anomalies remains to be seen. Of note, we previously showed that palliative use of sirolimus in diffuse cerebral proliferative angiopathy, which has multiple imaging and clinical similarities to bAVMs, reduced the number of ischemic attacks and significantly improved the quality of life for a pediatric patient [211].

### ANGPT2 inhibitors

Angiopoietins (ANGPT, or ANG) and their receptor tyrosine kinase, TIE2, are key regulators of angiogenesis and vascular stability. Receptor binding by the TIE2 agonist ANGPT1 promotes EC-pericyte interactions and vascular stability via activating TIE2. Conversely, ANGPT2 may repress TIE2 activity and destabilize EC-pericyte interactions, depending on the context [212]. Previous work from the Meadows lab showed that SMAD4 loss led to decreased TIE2 expression and elevated ANGPT2 expression in the postnatal murine retina, and that administration of LC10, an ANGPT2 blocking antibody, prevented AVM formation and normalized vessel diameter within the postnatal retinas of *Smad4*^*iECKO*^ animals [95]. They extended these studies to show that three different murine HHT models (*Smad4*^*iECKO*^, *Alk1*^*iECKO*^, *Eng*^*iECKO*^) all featured increased *Angpt2* expression and concomitant downregulation of *Tie2*, as well as a transcriptional signature of increased angiogenesis and cell migration. Importantly, ANGPT2 inhibition improved HHT pathology and normalized the brain vasculature in these murine models [66]. However, other studies of human and murine HHT yield contradictory findings in terms of alterations in ANGPT2 expression [28, 86, 213–215]. Suggestively, human sporadic bAVM samples, similar to these lesions in the inherited syndrome HHT, also feature decreased TIE2 protein levels [216], as well as diminished levels of the TIE2 agonist ANGPT1, but increased transcript and protein levels of the TIE2 antagonist ANGPT2 [217]; trends which were generally confirmed in a recent scRNA-seq study of human sporadic bAVM samples [218]. While it remains to be seen whether ANGPT2 is similarly affected, and potentially targetable, in murine models of sporadic bAVM, this may represent a shared therapeutic vulnerability and common mechanism in inherited and sporadic bAVM.

### Cell cycle inhibitors

Loss of BMP9/10 signaling disrupts endothelial quiescence and leads to increased endothelial cell proliferation [65, 68, 78, 82, 93, 94, 104, 219]. Intriguingly, a study from our own group, as well as a preprint from another laboratory, demonstrated that BMP9/10 blocking antibody (BMP9/10 Abs)-treated mouse models of HHT2 exhibit increased expression of Cyclin Dependent Kinase (CDK) 4 and 6 in AVMs within the vasculature of the postnatal murine retina [220, 221]. CDK4 and 6 normally phosphorylate, and thus deactivate, the retinoblastoma protein Rb, which is essential for cell cycle progression from the G1 to the S phase, and thus cell division. Administration of the FDA-approved CDK4/6 inhibitors Palbociclib, as well as Ribociclib, attenuated this endothelial hyperproliferation and prevented AVM formation in the neonatal retina of BMP9/10 Abs-treated mice in both studies. Moreover, our work showed that CDK4/6 inhibitors prevented vascular dysplasia in the neonatal retina and brain of *Alk1*^*iECKO*^ mutants at P7, while Dinakaran and colleagues showed CDK4/6 inhibition rescued retina defects in *Eng*^*iECKO*^ mice and cerebrovascular defects in BMP9/10 Abs-treated neonates [220, 221].

This novel therapeutic insight leveraged previous studies showing that cell cycle dynamics are a key regulator of arteriovenous identity within the postnatal murine retina, mediated via a Notch1-Cxn37-p27 axis that promotes endothelial cell cycle arrest and quiescence in response to arterial sheer stress [222–224]. Given the overt toxicity of these drugs, as well as their limited BBB permeability, other selective CDK4/6 inhibitors, such as abemaciclib, which is BBB permeable and also FDA approved, may be of interest for treating bAVMs. However, given the extensive incidence of side effects with CDK inhibitors (e.g., nausea, anemia, leukopenia, thrombocytopenia, vomiting, etc.), the use of these drugs and the treatment duration, particularly in pediatric patients, will have to be weighed carefully against a patient’s symptoms and need.

### Targeting the RAS-MAPK pathway

RAS mutations are thought to make up 20% of all cancers, with activating KRAS mutations alone comprising 35% of lung, 45% of colorectal, and 90% of pancreatic cancers [164, 225]. Due to the high prevalence of activating RAS mutations in cancer, several approaches have been developed to modulate the RAS pathway. One such approach involves farnestyltransferase inhibitors (FTIs) which block localization of RAS to the inner cell membrane [226]. While these inhibitors have been shown to elicit tumor regression in MMTV-v-HRAS mice, they have yet to induce significant tumor regression in KRAS driven tumors and have not been tested in bAVMs [227–230]. Selective covalent inhibitors targeting KRAS^G12C^, such as AMG-510 (sotorasib), have also emerged to combat constitutively active KRAS, but are currently only approved as a second line treatment in patients with non-small cell lung carcinoma [231, 232]. However, the discovery of *KRAS*^*G12C*^ mutations in bAVM (although rare) [143] make these inhibitors relevant for future research. Intriguingly, while not bAVMs, 2 patients with facial AVMs and confirmed G12C mutations showed an excellent response to sotorasib, with reduction of symptoms within one month [167]. Notably, this same study showed reduction of G12C-driven bAVMs in a mouse model (*Cdh5-PAC-CreER*; *Kras*^*lox−stop−lox−G12C*^) where animals were treated with sotorasib immediately after tamoxifen induction [167]. Relatedly, a recently described KRAS^G12D^ selective inhibitor, MRTX-1133 [233], may also prove useful in treating bAVM, given the frequency of this variant in sporadic bAVM [141, 142, 146]. Targeted protein degradation via PROTACS, or PROteolysis TArgeting Chimeras, also hold promise for bAVM treatment, with a designed ankyrin repeat protein (DARPin) molecule showing success in selectively impacting KRAS and KRAS^G12D^ function in cultured cells, without affecting activity of NRAS or HRAS [234]. Additionally, the BRAF^V600E^-specific inhibitor Dabrafenib prevented lethality and bAVM formation in AAV-BR1-Cre transduced Braf^V600E^ mice, but did not rescue established lesions in adult animals [177].

Finally, MEK inhibitors show promise in disrupting KRAS mutant driven bAVMs, as constitutively active KRAS appears to act predominantly through MEK1/2 in the endothelium, rather than PI3K/AKT, to alter the CNS endothelium [135, 146]. MEK inhibitors have already proved successful in animal models, causing regression of established lesions and preventing bAVM hemorrhage in endothelial specific KRAS^G12V^ bAVM zebrafish model [146] and preventing bAVM formation in a AAV-based *Kras*^*G12V*^ bAVM adult mouse model [166]. Another study suggests that that *Kras*^*G12D*^-induced changes in cerebral angiogenesis in *Cdh5-PAC-CreER*; *Kras*^*G12D*^ mice can be normalized by treatment with trametinib (a MEK inhibitor), although they did not examine AVMs in this report [235]. Small clinical studies have begun to use MEK inhibitors in patients with AVMs with promising results, including decreased complications and size reduction [236–239]. Of note, we have begun the compassionate use of the FDA-approved MEK inhibitor trametinib in pediatric bAVM cases and observed slowed disease progression in a handful of patients, and treatment with a BBB-permeable MEKi normalized KRAS-induced changes in the cerebrovasculature and bAVM induction in *Slco1c1-CreER*;*Kras*^*lsl−G12D/*+^ adult mice [163]. Because MEK inhibitors should be effective against all *KRAS* variants, as well as somatic activating mutations in *BRAF* or *MAP2K1* (which combined conservatively account for at least 50% of all sporadic bAVM cases), future consideration should be given to employing trametinib or other FDA-approved MEK inhibitors as a frontline, non-surgical therapy for treating bAVM.

## Future directions and unanswered questions

Despite defining the genetic causes of HHT and many cases of sporadic bAVM, the field lacks a complete understanding of the molecular and cellular dynamics driving the formation, growth and stability of these complex vascular anomalies. Continued refinement is needed to understand how inherited and somatic genetic variants promote both cell autonomous and non-autonomous effects on nidus formation and stability. Additionally, whether well-established bAVMs can be reversed using pharmacological approaches remains unknown. Furthermore, fundamental questions remain regarding where and when a mutation must be acquired, and whether multiple “hits”, are necessary for bAVM initiation and/or pathologic remodeling. Studies in preclinical vertebrate animal models, such as mice, should provide needed insights, as the development of arterial, venous, and capillary-restricted Cre drivers, as well as subpopulation-specific recombinase tools, or optogenetically controlled site-specific recombinases, will allow us to refine our understanding of the cellular origin of somatic bAVMs in vivo. The use of spatial transcriptomics and genome sequencing may also allow pathologists to trace the origin and timing of variant acquisition within patient samples.

Understandably, the field has focused predominantly on the role of the endothelium in the genesis and progression of bAVMs. However, with evidence mounting for the second hit hypothesis in HHT, and single cell data suggesting that non-endothelial populations play a critical role in communicating with the endothelium and driving intracerebral hemorrhage in bAVM [218, 240], much work remains to be done examining the contribution of pericytes, astrocytes, neurons, microglia, and other cell types within the brain in these devastating vascular anomalies.

While our understanding of the somatic genetic drivers associated with brain AVMs is still in its infancy, targeted medical therapies may yet be identified that can affect bAVM growth, development, or hemorrhage, which would allow for more personalized and non-invasive treatment options. Similar to recent advances in oncology, therapies tailored to the genetic and molecular signature of an individual anomaly may prove effective for bAVM treatment in the future. Identification of the somatic mutations in a bAVM could be achieved with “liquid” biopsy using endovascular catheters and coils to obtain endothelial cells from an AVM for genetic testing [241]. Accordingly, personalized therapies could be leveraged against the unique genetic and molecular profile of a bAVM.

Finally, we would highlight that each of the medications mentioned in this review have only been studied in isolation as monotherapies, and mostly in isolated cases or small cohorts based on compassionate use, and not large placebo-controlled clinical trials. Decades of work in oncology has shown that combinatorial treatment modalities routinely offer greater efficacy in terms of continued response and delayed therapeutic resistance. Going forward, clinical trials for bAVMs must embrace this same approach. Given the modest number of bAVM patients even at large treatment centers, multi-site clinical trials will be essential for determining the efficacy of such novel therapeutic approaches. The recent gains in our understanding of the genetic drivers of inherited and sporadic bAVM, as well as current advances in endovascular surgical approaches, suggest we may be at the precipice of a revolution in the care and management of asymptomatic and symptomatic bAVM, driven in no small part by preclinical studies in the mouse.

## Data Availability

No datasets were generated or analysed during the current study.
